# RAFT-ports: an application for risk assessment for tsunamis in ports demonstrated at Tanjung Gerem Port, Indonesia

**DOI:** 10.1186/s40562-026-00522-w

**Published:** 2026-11-07

**Authors:** Raquel Felix, Constance Ting Chua, Anawat Suppasri, Ignatius Ryan Pranantyo, Endra Gunawan, Mohammad Heidarzadeh

**Affiliations:** 1 Department of Architecture & Civil Engineering, University of Bath, Bath, Somerset BA2 7AY UK; 2 International Research Institute of Disaster Science (IRIDeS), Tohoku University, Sendai, 980-8572 Japan; 3 Center of Excellence in Disaster and Risk Management Information Systems, Department of Industrial Engineering, Faculty of Engineering, Chulalongkorn University, Bangkok, 10330 Thailand; 4 Earth Observatory of Singapore, Nanyang Technological University, 639798 Singapore, Singapore; 5 Faculty of Mining and Petroleum Engineering, Institut Teknologi Bandung, Kota Bandung, 40132 West Java Indonesia; 6 Hydro-Coast Consulting Engineers Ltd., Bath, Somerset BA2 2TB UK

**Keywords:** RAFT–ports, Tsunami, Tsunami impact assessment, Indonesia, Port structures, Seaport

## Abstract

The development of tsunami fragility curves and vulnerability functions has expanded in recent years, including those specifically for port environments. Given the importance of ports in global trade and supply chains, there is a need for operational tools that can support the assessment of potential tsunami impacts on port structures. In this study, we contribute to bridging the gap between published tsunami fragility curves and vulnerability functions and their practical application through the development of RAFT–Ports (Risk Assessment For Tsunamis in Ports). RAFT–Ports is an open-source, user-friendly application that applies fragility and vulnerability functions developed for port structures to estimate structural-damage exceedance probabilities, expected physical loss, and production-capacity recovery probabilities for specified tsunami scenarios. Its output is a report for scenario-based tsunami impact assessment, summarising damage, loss, and recovery metrics through inundation maps, bar charts, summary tables, and the complete set of raw analysis files. It is designed to be adaptable, allowing users to modify fragility-curve and vulnerability-function values as new empirical data become available. We demonstrate its functionality through a case study at Tanjung Gerem Port, Indonesia, using hypothetical M_w_ 8.9, M_w_ 8.7, and M_w_ 8.5 earthquake scenarios along the Sunda Megathrust. This work contributes to technology transfer between scientific research and end users by showing how fragility-curve and vulnerability-function data can be transformed into actionable assessments of tsunami impacts that can support risk-informed mitigation, preparedness, and recovery strategies.

## Introduction

Research on tsunami impacts to critical infrastructure has increased in recent years (e.g., Rodwell et al. 2023; Williams et al. 2024), particularly for seaports, which are highly exposed to coastal hazards and play a central role in global supply chains. Beyond direct physical damage, tsunami-induced port disruptions can generate cascading impacts across maritime transport networks by affecting shipping routes, trade flows, and the operational functions of neighbouring and distant ports. For example, ports along the west coast of the United States experienced damage and disruption from the 2011 Tohoku tsunami, resulting in substantial economic consequences despite their far-field location relative to the tsunami source (Rose et al. 2016). More recently, Chua et al. (2024) demonstrated how tsunami impacts can alter the connectivity and functionality of the global port network as vessels are rerouted to alternative ports following port closures. Consequently, fragility and vulnerability functions have been developed for a range of seaport applications, including structural damage, economic loss, and post-earthquake recovery (Chua et al. 2021; Miki et al. 2025; Suppasri et al. 2022). Estimates derived from these depth-based impact functions can provide valuable first-order information for understanding potential tsunami impacts at individual ports and supporting operational decision-making.

In this study, we aim to advance operational tsunami impact assessment by making fragility-curve approaches practically implementable for port environments through the development of an open-source, user-friendly, offline application with a graphical user interface. The application, termed RAFT–Ports (Risk Assessment For Tsunamis in Ports), is the first openly available tool designed to assess tsunami-induced structural damage in port facilities while also providing analyses of loss ratios and production-capacity recovery. It generates inundation maps, summary tables, and graphical outputs to support scenario-based impact assessments in ports. The application is adaptable, allowing users to update vulnerability information as new or improved fragility relationships become available. Unlike existing tsunami risk assessment platforms that primarily provide pre-generated products for predefined regions (Supplementary Table S1), *RAFT–Ports* enables site-specific assessments using user-defined inundation datasets, fragility and vulnerability functions, and structure inventories. Although it does not explicitly model network-scale disruptions, its outputs can support subsequent evaluations of port operability, economic losses, supply-chain effects, and broader maritime network impacts.

**Fig. 1:**
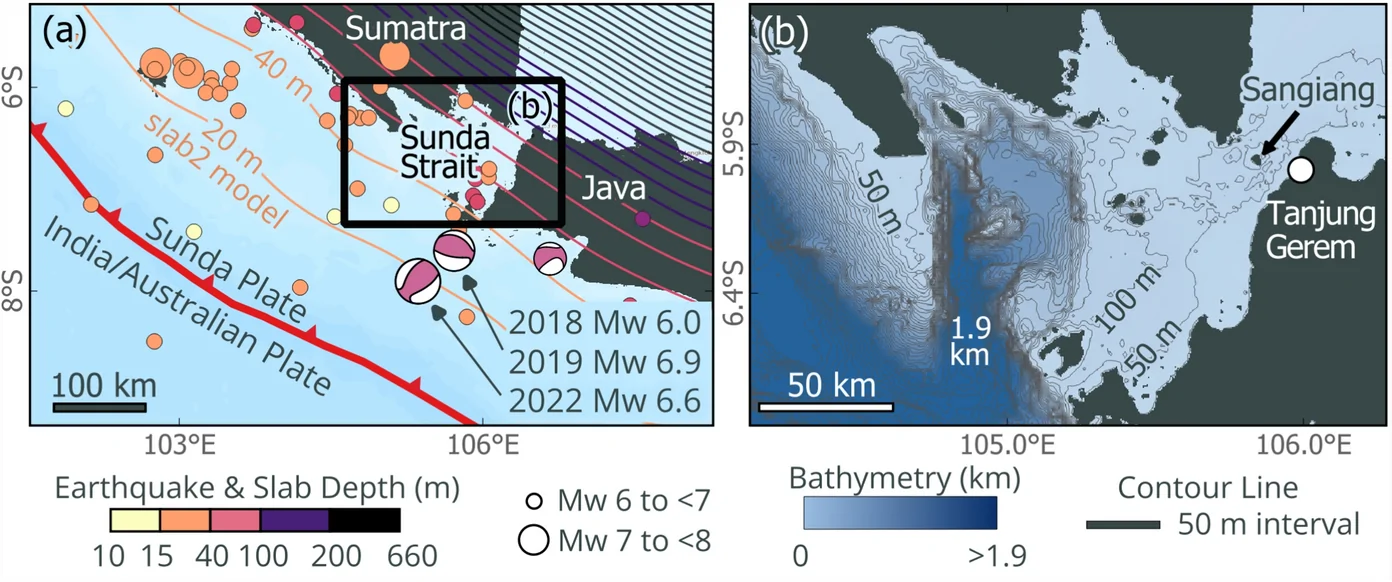
(**a**) Tectonic map showing the Java Trench of Indonesia (red), bathymetric contours of the subducting Indian/Australian Plate (Slab2; Hayes et al. 2018), and epicentres of M_w_6–8 earthquakes in the region in the period 1900-2025 (orange circles, Geological 2025). Focal mechanisms of some of the large earthquakes are shown based on Global CMT catalogue (Dziewonski and Anderson 1981; Ekström et al. 2012). Black rectangle shows the extent of the map on (**b**). (**b**) Bathymetry of the Sunda Strait showing a 1.9 km-deep submarine valley at its southwest entrance and *≤ *150 m depths elsewhere. The study area, Tanjung Gerem Port, lies on the northwest coast of Java Island

**Fig. 2:**
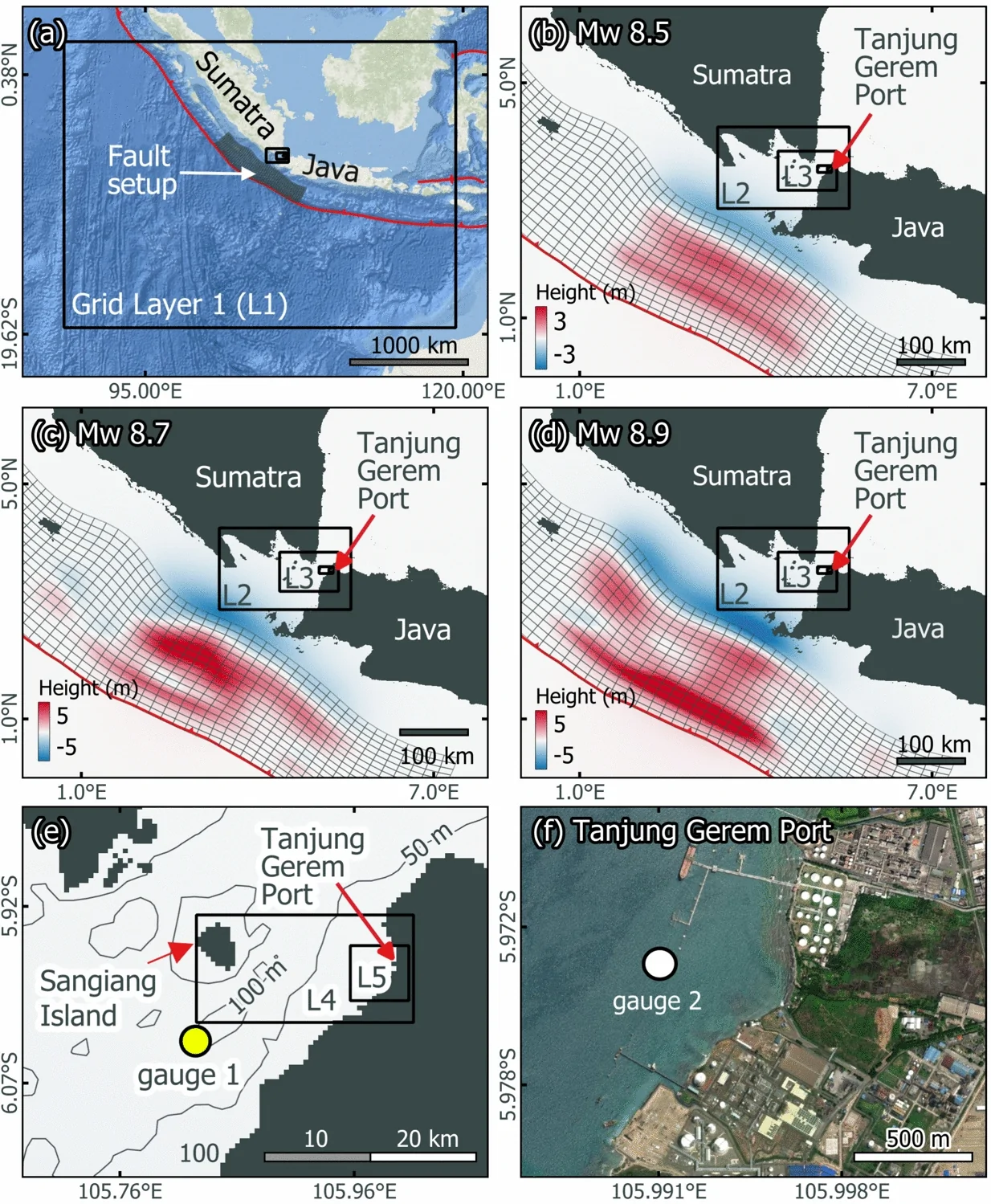
Fault plane and initial tsunami setup used in the numerical modelling. (**a**) Extent of the gridded fault model used to generate the slip distributions, covering the identified seismic gap, together with the extent of the outermost numerical grid (Layer 1). (**b**–**d**) Initial tsunami conditions for the selected worst-case M_w_8.5, M_w_8.7, and M_w_8.9 scenarios. (**e**) Extent of the two finest nested grids (Layers 4 and 5). (**f**) Satellite image of the study area, Tanjung Gerem Port, within the Cilegon Industrial Zone (World Imagery; Esri 2025)

To demonstrate the functionality of *RAFT–Ports*, we perform a scenario-based tsunami impact assessment at Tanjung Gerem Port, Java, Indonesia (Figs. 1 and 2), using numerically modelled tsunamis generated by hypothetical earthquake scenarios along the Sunda Megathrust with moment magnitudes (M_w_) of 8.5, 8.7, and 8.9 (Figs. 1a and 2a–d). Although the fragility curves used in this study are originally developed from observations of port and industrial facility damage during the 2011 Tohoku tsunami in Japan, they remain the only comprehensive and publicly accessible set of curves available for port-related tsunami impacts. Given this limitation, their application to Tanjung Gerem Port should be interpreted as an optimistic lower bound of potential risk because of differences in construction practices, structural characteristics, and local port configurations between sites in Japan and Indonesia.

## Sunda strait and sunda megathrust

The Sunda Strait is bounded by Sumatra to the northwest and Java to the southeast (Fig. 1a). The dominant bathymetric feature of the strait is a submarine valley at its southwestern entrance, which occupies nearly half its width and reaches a maximum depth of ~ 1.9 km (Fig. 1b). At the northeastern entrance, Sangiang Island divides the strait into two narrow channels. Our study area, Tanjung Gerem Port, is located along the coast facing the eastern channel (Fig. 1b).

Southwest of the the Sunda Strait lies the Sunda Megathrust (Figs. 1a and 2a), a 5000–km curvilinear fault formed by the subduction of the northeastward-moving India/Australian Plate beneath the Sunda Plate (Simons et al. 2007; Sieh 2007; DeMets et al. 2010). Several segments of this fault have hosted major tsunamigenic earthquakes, notably the 2004 M_w_9.1 Sunda–Andaman earthquake (northern segment; e.g., Vigny et al. 2005; Lay et al. 2005; Rhie et al. 2007), the 2006 M_w_7.7 tsunami earthquake off Java (e.g., Ammon et al. 2006; Fritz et al. 2006; Mori et al. 2007; Gunawan et al. 2016), and the 2010 M_w_7.8 tsunami earthquake near Mentawai Island (e.g., Hill et al. 2012; Satake et al. 2013). The closest tsunami-generating segment to the study area generated the 2006 Java tsunami earthquake. It is located off the southwestern coast of Java, ~ 500 km west of the fault segment examined in this study.

The segment of the Sunda Megathrust southwest of the Sunda Strait (Fig. 2a) has not generated an earthquake larger than M_w_7 since the 1960s; recent relatively strong earthquakes in this region include the 2018 M_w_6, 2019 M_w_6.9, and 2022 M_w_6.6 events with focal depths of 40-55 km (focal mechanism solutions shown in Fig. 1a). Therefore, this segment of the Sunda Megathrust is considered a seismic gap (Fig. 2a), based on analyses of earthquake catalogues (International Seismological Centre 2025; Dziewonski and Anderson 1981; Ekström et al. 2012) and previous studies (Hanifa et al. 2014; Widiyantoro et al. 2020). It has been estimated to be capable of generating a M_w_ 8.9 earthquake, which could potentially produce a significant tsunami. Consequently, assessing the potential tsunami impacts associated with this seismic gap is essential.

While probabilistic tsunami hazard assessments have been conducted for the Sunda Megathrust, most focus on general hazard mapping along the Indonesian coasts (e.g., Horspool et al. 2014). Only the studies by Widiyantoro et al. (2020), Supendi et al. (2023), and Sriyanto et al. (2024) provide more localised analyses examining tsunami hazards associated with the seismic gap along the southern coasts of eastern Sumatra and western/central Java. Among these works, Supendi et al. (2023) and Sriyanto et al. (2024) include tsunami sources that extend into the Sunda Strait, the region of interest in this study. Supendi et al. (2023) performed deterministic modelling using a uniform slip distribution, focusing on tsunami impacts along Java’s southern coast. Sriyanto et al. (2024) conducted deterministic modelling of an M_w_ 8.9 scenario, including inundation modelling for the Cilegon industrial zone, and employed both uniform and non-uniform slip distributions to compare inundation results using different elevation models. Although Sriyanto et al. (2024) conducted inundation modelling covering our case study site, partly overlapping with the scope of our work, our study provides a more comprehensive assessment of potential tsunami impacts on Tanjung Gerem.

## Methodology

### Fault setup

We used the extent of the seismic gap defined by previous studies (Hanifa et al. 2014; Supendi et al. 2023; Sriyanto et al. 2024), to delineate the earthquake source region (Fig. 2a). This delineation represents the total possible rupture area for a M_w_ 8.9 earthquake and is therefore set as the largest earthquake scenario used in our tsunami modelling. To explore how tsunami impact varies with M_w_, we also included hypothetical scenarios of M_w_ 8.7 and M_w_ 8.5. For each magnitude, we generated 100 stochastic slip models by varying earthquake parameters, resulting in a total of 300 slip models for this study (Supplementary Figures S1–S3). Summary statistics of the 100 stochastic slip realisations for each earthquake magnitude are presented in Supplementary Table S2 and Figure S4. Mean slip values range from 4.84 to 9.87 m, with standard deviations of 1.62 to 3.35 m, while mean fault-plane areas range from 47,385 to 90,440 km^2^, with standard deviations of 16,284 to 26,084 km^2^. Although only the scenario producing the largest inundation extent was selected for the *RAFT-Ports* demonstration, these statistics provide an indication of the variability associated with the stochastic source generation.

The fault plane configuration and slip distribution are generated following Pranantyo et al. (2026), who applied the Probabilistic Tsunami Hazard Assessment (PTHA) package developed by Geoscience Australia (Davies et al. 2015, 2017; Davies and Griffin 2020; Australia 2018). First, the SLAB2.0 subduction interface model of Hayes et al. (2018) is discretised into quasirectangular unit sources of 20 km × 10 km (Fig. 2a–d). Seafloor deformation for each unit source is then computed using the analytical solutions of Okada (1985), applying a uniform slip of 1 m. The rupture area for a given M_w_ is estimated using the empirical scaling relationships of Strasser et al. (2010) for subduction interface, with rigidity set to 30 GPa. Heterogeneous random slip distributions are generated using the stochastic k-2 slip formulation described by Davies et al. (2015). In this approach, the slip field follows the standard k^2^ spectral method of Gallovič and Brokešová (2004), with any negative slip values clipped to zero, and the final slip pattern smoothed using a Gaussian filter.

The initial seafloor deformation is obtained by linearly superposing the heterogeneous slip model with the pre-computed unit-source deformations. This deformation is then used as the initial tsunami condition, under the assumption that the tsunami is generated instantaneously with the earthquake (Heidarzadeh et al. 2025). This assumption is appropriate here because the fault dimensions, spanning hundreds of kilometres, are much greater than the maximum local water depth at the source region (~ 6 km), making water column filtering effects negligible (Kajiura 1963).

### Tsunami modelling

Tsunami propagation and inundation are modelled using the open-source FUNWAVE–TVD v4.6 model (Kirby et al. 1998; Wei et al. 1995; Shi et al. 2012). Input preparation and visualisation are performed using the FUNMAP applications (Felix et al. 2025), and simulations are run on the Nimbus cloud supercomputer at the University of Bath. As the primary aim of this study is to demonstrate the functionality of *RAFT–Ports*, only the worst-case inundation scenario is presented for each earthquake magnitude. To identify this scenario, 100 stochastic slip models are simulated for each M_w_ using the coarsest grid (Layer 1 in Fig. 2a; Supplementary Table S3), and the model producing the largest nearshore wave amplitude at a virtual gauge (Supplmentary Figure S5) near Tanjung Gerem Port (Fig. 2e) is selected.

The coarse-grid bathymetry is derived from GEBCO 2025 (GEBCO 2025) and resampled to approximately 1,280 m. A five-level nested-grid system with a 4:1 refinement ratio is used to achieve the 5 m grid resolution required for inundation modelling and subsequent impact assessment (Fig. 2a–e; Supplementary Table S3). Although Felix et al. (2024) showed that 10–50 m bathymetric grids can reproduce coastal wave heights comparable to those obtained from a 5 m grid, the finer resolution is adopted here to adequately represent port-scale features such as narrow jetty piles and bridges. For the nested-grid simulations, bathymetry is constructed from BATNAS and BIG’s Coastal Environment Map, while topography is obtained from FABDEM v1.2. All datasets are processed in QGIS v3.44 (Qgis Development Team 2026) and referenced to Mean Sea Level (MSL).

In the first three grid layers (Fig. 2a–d), we employed the linear shallow-water equations and switched to the nonlinear shallow-water equations in grid layers 4 and 5 (Fig. 2e). The bottom-friction coefficient used in layers 4 and 5 is 0.025, and the Courant number (Courant et al. 1928) is 0.25. The total simulation time is set to 5 hours, as our initial tests indicated that this duration is sufficient to capture multiple wave arrivals, with the largest waves at the port typically occurring within the first three hours. A virtual gauge is placed at the 10 m depth contour near Tanjung Gerem Port (Fig. 2f) to show the tsunami wave pattern through time for the three worst-case scenarios associated with the M_w_ 8.9, M_w_ 8.7, and M_w_ 8.5 earthquakes.

### Study area and building classifications

Almost the entire Cilegon coastline is developed for industrial purposes. To narrow the study area for testing *RAFT–Ports*, we selected a smaller area within Tanjung Gerem Port based on the following criteria: (a) the presence of at least two industry types; (b) well-defined buildings in satellite imagery; (c) proximity to the coastline; and (d) previous identification as an area extensively affected by inundation (Sriyanto et al. 2024).

Building footprints are obtained from OpenStreetMap (OpenStreet 2025) and verified in QGIS (Qgis Development Team 2026) against Google Satellite Hybrid imagery, with missing buildings manually delineated, resulting in a dataset of 430 buildings. The attribute table of the shapefile is updated to include industry-type classifications following Chua et al. (2021), based on company names from OpenStreetMap land-use data and Google Satellite Hybrid imagery. Of the 430 buildings, 346 are classified as Chemical and 84 as Petrochemical. The resulting shapefile and modelled inundation file are used as inputs to *RAFT–Ports*.

### Application development

The *RAFT–Ports* is developed using MATLAB App Designer R2024b. Microsoft 365 Copilot is used to support coding, improve development efficiency, and assist in organising the code structure. Portions of the code and interface layout are adapted from Felix et al. (2025). It is freely available as standalone executable packages for Windows and macOS, requiring only the bundled MATLAB Runtime library, as well as a version that can be run directly within MATLAB. The GitHub repository containing the installers, source code, sample datasets, and user manual is listed in the ‘Code and Data Availability’ section of this paper.

**Fig. 3:**
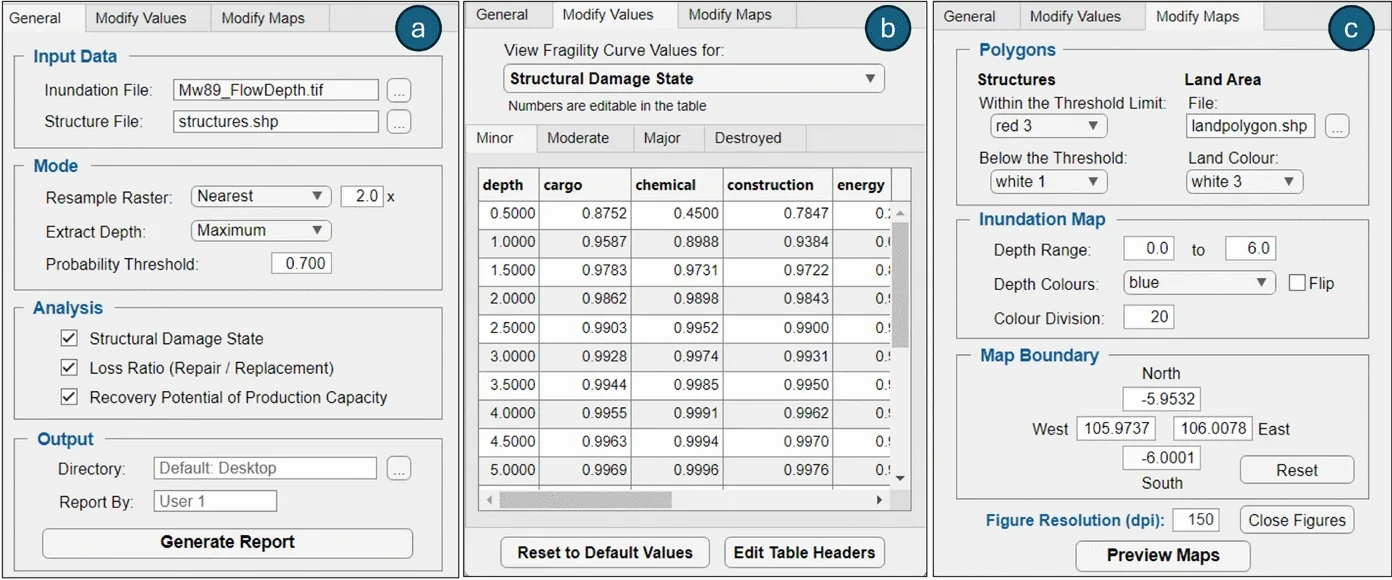
The *RAFT–Ports* graphical user interface (GUI) developed in this study. (**a**) General tab for entering all required inputs. (**b**) Modify Values tab for updating the reference values used in the analysis. (**c**) Modify Maps tab for adjusting map visualisation settings

The graphical interface consists of three tabs (Fig. 3). The ‘General’ tab contains the functions and settings required to generate the report (Fig. 3a). The ‘Modify Values’ tab allows users to adjust the fragility-curve parameters and values (Fig. 3b), while the ‘Modify Maps’ tab provides options for customising map visuals (Fig. 3c). Further details are provided in the user manual.

### Analysis in *RAFT–Ports*

The analyses require two input files: an inundation file (GeoTIFF format), assumed to be derived from tsunami modelling based on a digital surface model (DSM), and a building-footprint shapefile. The shapefile must include a ‘type’ field identifying the industry classification of each building (e.g., cargo, petrochemical industry), which is used to assign the appropriate fragility curve during the analysis.

**Fig. 4:**
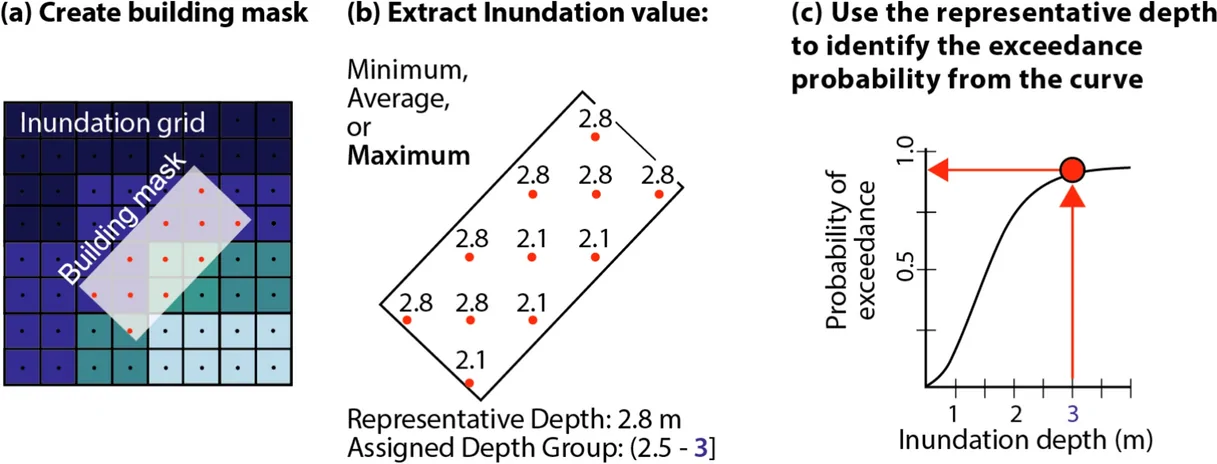
Simplified explanation of how *RAFT–Ports* extracts the analysis values for each building footprint

Inundation depths are extracted for each building using a masking function (Fig. 4a). Because a single depth value is required for subsequent analyses, *RAFT–Ports* assigns one representative inundation depth to each building footprint. Where multiple inundation values are present, the representative depth can be defined as the minimum, mean, or maximum value, with the maximum used by default (Fig. 4b). To further simplify the analysis, representative depths are assigned to a Depth Group at 0.5 m intervals. For example, a building inundated by 2.8 m is assigned a Depth Group of ‘(2.5–3]’ (Fig. 4b). The upper-bound value of the corresponding depth interval is then used to link the building to the appropriate fragility curve or vulnerability function and estimate the corresponding probability of exceedance (Fig. 4c). The use of 0.5-m inundation-depth intervals follows Adriano et al. (2014); however, unlike their approach, which uses the interval mean as the representative depth, *RAFT–Ports* adopts the upper-bound value.

Three types of impact analysis are available in *RAFT–Ports*: structural damage state, loss ratio, and recovery potential. The structural damage state and recovery-potential analyses are based on probability-of-exceedance values derived from depth-based fragility curves, whereas the loss-ratio analysis is based on expected loss ratios derived from vulnerability functions. By default, the application uses the average structural-damage fragility curves of Chua et al. (2021), the loss-ratio vulnerability functions of Miki et al. (2025), and the recovery-potential fragility curves of Suppasri et al. (2022) (Supplementary Tables S4–S6). Although inundation depth alone does not explicitly capture debris impacts, scour, or other tsunami loading mechanisms, the fragility curves are developed from observed damage resulting from the combined effects of depth and velocity, making them suitable for tsunami impact assessment. For the structural damage state analysis, we adopt the four damage-state categories of Chua et al. (2021): Minor, Moderate, Major, and Destroyed (DS1–DS4; Supplementary Table S7). The loss ratio represents the expected repair cost as a proportion of the full replacement cost and provides an estimate of functional loss. Unlike the structural damage state and recovery-potential analyses, the loss-ratio analysis uses expected loss ratios derived from vulnerability functions rather than direct probabilities of exceedance. Fragility curves are available for the following industry types: cargo, chemical, construction, energy, food, manufacturing, petrochemical, and warehouse structures, while the loss-ratio vulnerability functions additionally include transportation structures. The recovery potential analysis estimates the probability of a structure regaining its full pre-earthquake production capacity and accounts for indirect impacts (e.g., power shortages) that may affect both inundated and non-inundated structures (Suppasri et al. 2022). In our case study, we used the default reference values provided in the application. However, we emphasise that these values can be modified by the user within *RAFT–Ports*.

Because the analyses are derived from fragility and vulnerability functions, the results should be interpreted as scenario-based tsunami impact assessments rather than probabilistic or annualized tsunami risk estimates. For structural damage state, the reported damage level represents the minimum damage state expected to be exceeded. For example, the Minor damage in this study should be interpreted as at least Minor structural damage rather than exactly Minor damage, as the fragility curves are expressed as probabilities of exceedance. Similarly, Moderate, Major, and Destroyed damage states represent the probability of experiencing at least that level of damage. For loss ratio, the reported value represents the expected repair cost as a proportion of the full replacement cost. For recovery potential, the reported recovery period represents the possible earliest period at which the specified level of production-capacity recovery exceeds the selected probability threshold.

For simplicity, the *RAFT–Ports* report presents only the number of buildings at risk based on a user-defined threshold. For the structural damage state and recovery-potential analyses, this threshold corresponds to a probability-of-exceedance value, whereas for the loss-ratio analysis it corresponds to an expected loss-ratio value. By default, a threshold of 0.7 is applied, although this can be adjusted according to the user’s risk appetite, that is, the user’s tolerance to potential risk or loss. For the structural damage state and recovery-potential analyses, a threshold of 0.7 corresponds to a 70% probability of exceedance. For example, a probability-of-exceedance value of 0.7 for the Minor Damage state indicates a 70% probability that a structure will experience Minor damage or a more severe damage state. For the loss-ratio analysis, a threshold of 0.7 corresponds to an expected loss ratio equal to 70% of the full replacement cost. Structures meeting or exceeding the selected threshold are counted and reported in the output. In addition to the report, *RAFT–Ports* generates raw output files, including a building-footprint mask (.tif), image files (.png), data tables (.csv), and an updated building-footprint attribute table (0_Polygon_Masterfile.csv).

**Fig. 5:**
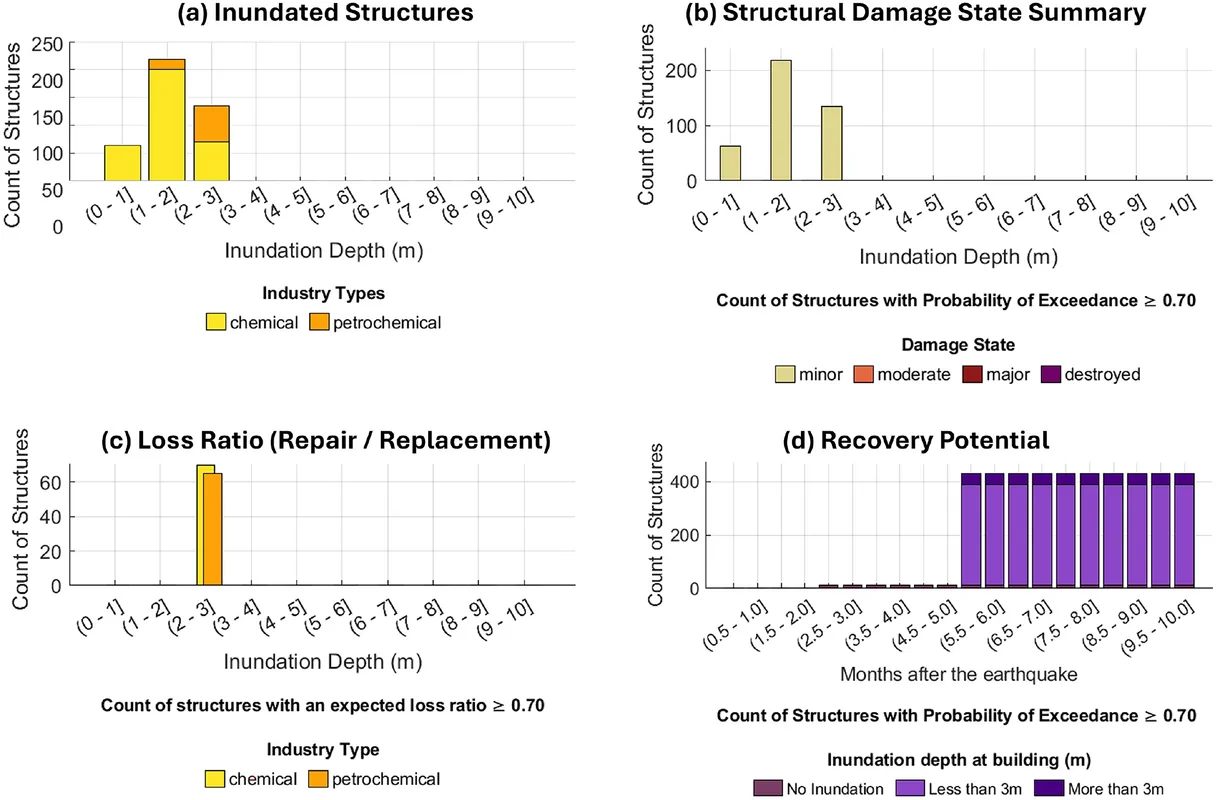
Bar-plot outputs generated by *RAFT–Ports* for the M_w_ 8.9 tsunami scenario, summarising the inundation, structural damage, loss ratio, and recovery potential analyses

To verify the implementation of the methodology in *RAFT–Ports*, the application outputs are compared with those obtained from an independently replicated workflow using QGIS and Excel. The validation focuses on the core analytical workflow, including inundation-depth extraction, DepthGroup assignment, and subsequent impact calculations. Across multiple combinations of inundation-depth extraction methods and raster-resampling settings, differences in DepthGroup assignment remain minimal (*≤ *2.3%), while independent probability-of-exceedance calculations performed in Excel produced identical results to those generated by *RAFT–Ports* (Supplementary Table S8 and Figures S6–S7). These findings demonstrate that the application correctly reproduces the intended analytical workflow for the datasets examined. Although the agreement on the actual value comparison between RAFT–Ports and the manual calculation is strong for the present case study, this does not necessarily imply that similarly small differences will occur in all applications. Rather, the validation should be interpreted as a cross-verification exercise that users can independently replicate for their own datasets and study areas. Its primary purpose is to confirm that the core workflow implemented in *RAFT–Ports* is functioning as intended. Additional sensitivity analyses show that the choice of user-defined threshold can influence the number of structures reported within the structural damage, loss-ratio, and recovery-potential categories, with lower threshold values increasing the number of structures classified as exceeding the specified damage, loss, or recovery criteria (Supplementary Table S9 and Figures S8–S10).

## Scope and applicability

Tsunami damage is not governed solely by inundation depth, but is also influenced by flow velocity, hydrodynamic and hydrostatic forces, buoyancy effects, debris impacts, and foundation scour. Consequently, inundation depth alone cannot explicitly represent all tsunami loading and failure mechanisms, particularly in port environments where velocity-dependent forces and debris impacts may be important. Furthermore, structural damage may occur before maximum inundation depth or flow velocity is reached (Suppasri et al. 2019). Despite these limitations, inundation depth remains the most consistently available and reliable parameter derived from post-tsunami field surveys and has therefore been widely used in the development of empirical tsunami fragility functions for port structures. In this context, inundation depth acts as a practical surrogate for multiple tsunami loading mechanisms that are implicitly reflected in the damage states proposed by Chua et al. (2021). Accordingly, *RAFT–Ports* adopts a depth-based approach for scenario-based tsunami impact assessment. The resulting estimates should be interpreted as preliminary and may underestimate impacts where complex failure mechanisms are significant.

Another limitation of *RAFT–Ports* is its dependence on the availability of building-footprint data and industry-type information for the structures being assessed. For data-poor ports, building footprints may need to be manually digitised using openly available satellite imagery. In QGIS, the ‘Basemaps’ plugin provides access to at least 9 available satellite-imagery sources, including Google Satellite Hybrid, which can display company names and assist in verifying building footprints and assigning industry-type classifications. In many cases, freely available building-footprint shapefiles from sources such as OpenStreetMap can provide a useful starting point, although these datasets should be reviewed and updated before being used in the analysis.

## Results

### Tsunami behaviour in the sunda strait

**Fig. 6:**
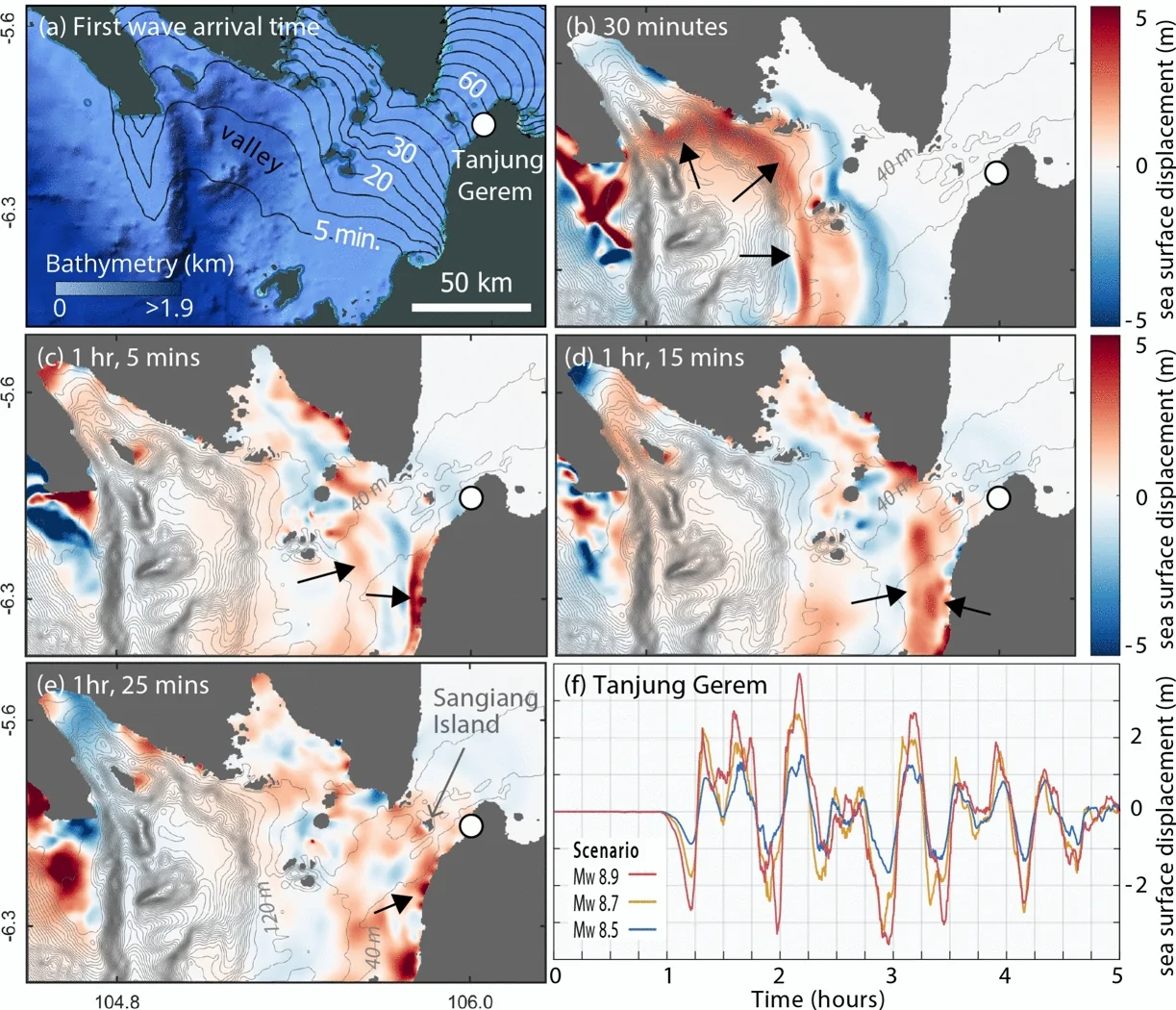
Numerical tsunami-model outputs for the Sunda Strait. Panels (**a**) to (**e**) are snapshots from the M_w_ 8.9 scenario. (**a**) Arrival-time contour of the first wave, defined as the time when the modelled sea surface first exceeds 0.1 m above mean sea level. (**b**–**d**) Tsunami heights at selected time snapshots for the M_w_ 8.9 scenario, with time after the earthquake. (**f**) Wave pattern recorded by the virtual gauge station (white dot) located at the 10 m depth contour near Tanjung Gerem for all three earthquake scenarios

The tsunami propagates into the Sunda Strait within five minutes of the earthquake (Fig. 6a). The submarine valley induces fan-shaped wave propagation, with slower wave speeds over the deep valley (wider contour spacing at the valley, Fig. 6a). and higher wave amplification along its flanks (Fig. 6b), consistent with previous submarine canyon studies (Iglesias et al. 2014; Felix et al. 2025). Beyond the valley, the wave front splits into three branches, two directed towards Sumatra and one towards Java (as pointed by the arrows in Fig. 6b). As the wave approaches the northeastern opening of the strait (Fig. 6c–d), Sangiang Island further channels the flow into two narrow passages (Fig. 6e), with Tanjung Gerem Port facing the eastern passage.

Time-snapshot outputs indicate that waves arriving at Tanjung Gerem Port are influenced by reflection and refraction along the concave Java coastline before entering the eastern passage (as pointed by the arrows in Fig. 6c–e). Within the port, jetty piles further modify wave behaviour through local refraction, wave entrapment in shallow coastal areas, and amplification near the shoreline (Supplementary Figure S11).

The virtual gauge station at the port entrance (gauge 2 in Fig. 2f) shows a similar sequence of wave arrivals for the M_w_ 8.9, M_w_ 8.7, and M_w_ 8.5 scenarios, differing mainly in wave height (Fig. 6f). An initial sea-level drawdown occurs approximately 56 min after the earthquake, followed by wave peaks at 1.31 h, 1.59 h, and 2.17 h. The third wave is the largest, reaching heights of 3.7 m, 2.17 m, and 1.54 m for the M_w_ 8.9, M_w_ 8.7, and M_w_ 8.5 scenarios, respectively. Although elevated waves continue thereafter, the maximum wave height and inundation extent appear to be reached within the first 2–3 h, with no further increase observed in the 3-, 4-, and 5-h simulation outputs.

### Tanjung gerem port

**Fig. 7:**
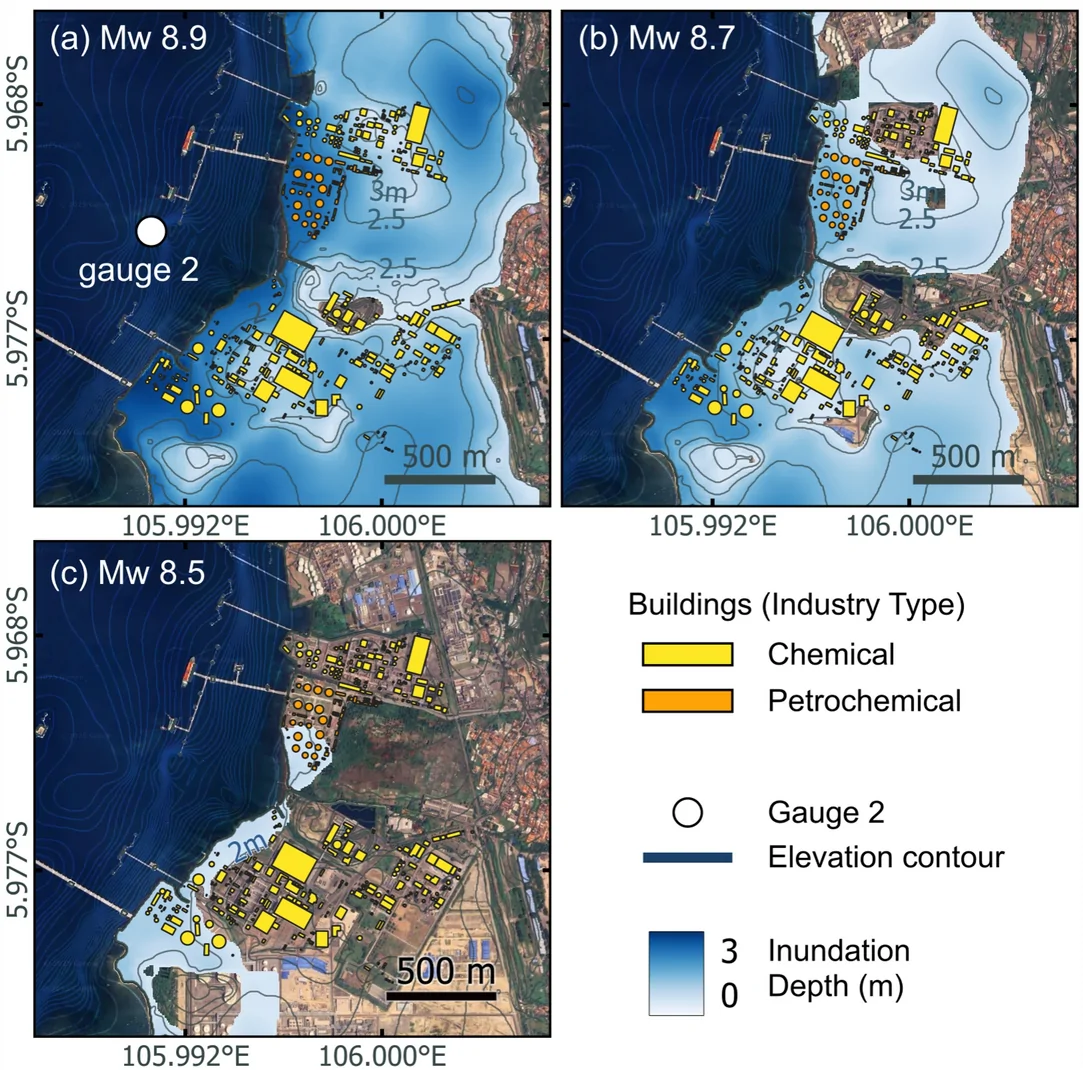
Inundation-modelling outputs and building footprints for the three earthquake scenarios. The maps show the maximum inundation depths simulated for the (**a**) M_w_ 8.5, (**b**) M_w_ 8.7, and (**c**) M_w_ 8.9 earthquake scenarios. These inundation datasets and building footprints serve as inputs to the subsequent *RAFT–Ports* impact assessment

Across all three impact metrics, estimated tsunami impacts at Tanjung Gerem Port increase with earthquake magnitude, with the M_w_ 8.9 scenario generating the deepest inundation depths and largest inundation extent (Fig. 7). Consequently, this scenario is associated with the largest proportion of affected structures, followed by the M_w_ 8.7 and M_w_ 8.5 scenarios (Table 1). Using a probability-of-exceedance threshold of 0.70, 73%, 13%, and 0% of structures in the M_w_ 8.9, M_w_ 8.7, and M_w_ 8.5 scenarios, respectively, are associated with at least the Minor damage state. These percentages represent structures with a *≥ *70% probability of experiencing Minor damage or a more severe damage state. Consistent with these generally low damage estimates, only 31%, 20%, and 0% of structures in the M_w_ 8.9, M_w_ 8.7, and M_w_ 8.5 scenarios, respectively, exceed the selected expected loss-ratio threshold. Using a probability-of-exceedance threshold of 0.70, some structures are projected to achieve the specified level of production-capacity recovery within approximately 2–2.5 months after the tsunami, while all structures reach at least a 70% probability of achieving the same recovery level within 5–5.5 months.

These relatively modest impacts may reflect both site-specific conditions and limitations in the available topographic and bathymetric datasets used in the tsunami simulations, particularly the topographic data. In particular, limitations in the horizontal and vertical resolution of the raw topographic data, together with uncertainties in its vertical accuracy, may not fully capture local-scale features that influence inundation patterns and depths, potentially resulting in underestimated tsunami impacts in some areas. These limitations of the inundation modelling provide further support for interpreting the results of this study as a likely lower-bound estimate of tsunami impacts, alongside the transferability uncertainties associated with applying fragility functions derived from observations of the 2011 Tohoku tsunami to a study area in Indonesia. Nevertheless, the results remain useful for identifying areas that may be more vulnerable to tsunami hazards and should therefore be prioritised for future detailed investigations using higher-resolution datasets.

**Table 1:** Summary of structural damage state, loss ratio, and recovery potential (in %) for the 430 assessed structures. A threshold value of 0.70 is applied to identify the structures included in the counts. For the structural damage state and recovery-potential analyses, this corresponds to a probability of exceedance of at least 70%, while for the loss-ratio analysis it corresponds to an expected loss ratio of at least 70% of the replacement cost. A detailed breakdown of the results for each industry type is in Supplementary Tables S10 and S11

All Structures, n = 430				
Scenario	Structural damage state (%)	Loss ratio (%)	Recovery potential (%)	
	Minor		2–2.5 months	5–5.5 months
M_w_ 8.9	97	31	3	100
M_w_ 8.7	73	20	18	100
M_w_ 8.5	13	0	85	100

## Conclusions

In our case study at Tanjung Gerem Port, the *RAFT–Ports* provides a consistent and efficient framework for analysing and presenting potential tsunami impacts. By presenting the results in a uniform format across all buildings and risk metrics, the tool enables the rapid identification of structures with elevated damage probabilities and expected loss ratios, and longer recovery times. Using a threshold value of 0.70, the analysis indicates that many structures have a high probability of exceeding the Minor damage state, exhibit elevated expected loss ratios, and are expected to experience extended recovery periods, with all structures showing prolonged recovery durations. Such information can help port managers and decision-makers prioritise facilities for detailed engineering assessments, resilience-enhancement measures, emergency planning, and post-disaster recovery efforts. These findings can also support proactive risk-reduction strategies and investment decisions aimed at improving the resilience of critical port structures to future tsunami events.

The Risk Assessment For Tsunamis in Ports (*RAFT–Ports*) provides an initial scenario-based tsunami impact assessment using depth-based fragility curves for structural damage and recovery analyses, and vulnerability functions for loss-ratio estimation. Its use is scalable and can be applied to larger study areas and other ports, provided that the user has the necessary input files. As the underlying fragility-curve and vulnerability-function values are editable, it can be adapted to alternative depth-based impact functions. Although this first version does not support multivariate fragility functions, the availability of the source code enables further development by both the authors and other researchers, including the addition of features such as ship-mooring analyses and multi-hazard assessments. *RAFT–Ports* provides a practical framework for incorporating published fragility curves and vulnerability functions into user-friendly, scenario-based tsunami impact assessments. Its adaptability supports the continued advancement of tsunami impact assessment methodologies, their application across a wide range of study areas, and the effective transfer of scientific knowledge and methods to end users and decision makers. As an initial version, *RAFT–Ports* focuses on addressing the key requirements of tsunami impact assessment in port environments and can serve as a key resource for translating fragility-curve research into operational assessment tools.

## Data Availability

The supplementary information and all associated resources are available at: https://github.com/raquelpfelix.

## Code availability

The *RAFT–Ports* installers, user manual, sample input and output files are available at: https://github.com/RAFT-Ports.
